# PLACENTAL MUTATIONAL BURDEN NEGATIVELY CORRELATES WITH GESTATIONAL AGE IN EARLY-ONSET PREECLAMPSIA

**DOI:** 10.1093/geroni/igad104.2492

**Published:** 2023-12-21

**Authors:** Hector Chavez, Nicholas Guo, Molly Huang, Morgan Meads, Omonigho Aisagbonhi, Mana Parast, Mariko Horii, Kathleen Fisch

**Affiliations:** University of California San Diego, San Diego, California, United States; University of California, San Diego, San Diego, California, United States; University of California, San Diego, San Diego, California, United States; UCSD, San Diego, California, United States; University of California, San Diego, San Diego, California, United States; University of California San Diego, San Diego, California, United States; University of California San Diego, San Diego, California, United States; University of California, San Diego, San Diego, California, United States

## Abstract

The placenta exists in a hypoxic environment during early pregnancy, making it susceptible to somatic genetic mutations caused by oxidative stress and rapid cell proliferation. Oxidative stress is one of the leading factors involved in cellular aging causing genetic mutations, cellular senescence, and mitochondria dysfunction over time. While it has been demonstrated that healthy placentas accumulate somatic mutations as they develop, we hypothesize that diseased placentas display an accelerated aging phenotype through the accumulation of somatic mutations. To address this, we analyzed RNA-seq data from 84 human placentas throughout gestation from healthy (N=33) and preeclamptic pregnancies (N=51). We identified rare single nucleotide variants, insertions and deletions using GATK Best practices Workflow for RNA-seq short variant discovery using bcbio-nextgen and annotated with VEP and LOFTEE. Mutational burden, defined as the total number of rare mutations per megabase, was calculated after strict quality control and filtering for rare (gnomADg< 0.05) variants using maftools in R. Linear regression models were employed to determine the association between mutational burden and gestational age. Differences in mean values were computed with a pairwise t-test . This study revealed that mutational burden in early-onset preeclampsia placentas is negatively correlated to gestational age (R^2=0.256; p-value=0.032). The early-onset preeclampsia group had a higher mutational burden relative to the other groups (p-value< 0.05). This study revealed a significant correlation of mutational burden with gestational age in early-onset preeclampsia, paving the way for additional investigation of accelerated aging in placentas and its contribution to the development of preeclampsia.

